# Duck enteritis virus UL54 is an IE protein primarily located in the nucleus

**DOI:** 10.1186/s12985-015-0424-z

**Published:** 2015-11-25

**Authors:** Chaoyue Liu, Anchun Cheng, Mingshu Wang, Shun Chen, Renyong Jia, Dekang Zhu, Mafeng Liu, Kunfeng Sun, Qiao Yang, Xiaoyue Chen

**Affiliations:** Avian Diseases Research Center, College of Veterinary Medicine, Sichuan Agricultural University, Wenjiang, Chengdu City, Sichuan 611130 P.R. China; Key Laboratory of Animal Diseases and Human Health of Sichuan Province, Wenjiang, Chengdu City, Sichuan 611130 P.R. China; Institute of Preventive Veterinary Medicine, Sichuan Agricultural University, Wenjiang, Chengdu City, Sichuan 611130 P.R. China

**Keywords:** Duck enteritis virus, UL54, Expression, IE, Intracellular localization

## Abstract

**Background:**

The UL54 protein of Duck Enteritis Virus (DEV) is a homolog of herpes simplex virus-1 (HSV-1) immediate-early infectious cell protein 27 (ICP27), a multifunctional protein essential for viral infection. Nonetheless, there is little information on the UL54 protein of DEV.

**Methods:**

The UL54 gene was cloned into the pPAL7 vector, and the recombinant protein, expressed in the E. coli Rosetta, was used to produce a specific antibody. Using this antibody, Western blotting and indirect immunofluorescence analysis (IFA) were used to analyze the expression level and intracellular localization, respectively, of UL54 in DEV-infected cells at different times. Real-time quantitative reverse transcription PCR (RT-PCR) and the pharmacological inhibition test were utilized to ascertain the kinetic class of the UL54 gene.

**Results:**

UL54 was expressed as a fusion protein of approximately 66.0 kDa using the prokaryotic expression system, and this protein was used to generate the specific anti-UL54 antibody. The UL54 protein was initially diffusely distributed throughout the cytoplasmic region; then, after 2 h, it gradually distributed into the nucleus, peaking at 24 h, and complete localization to the nucleus was observed thereafter. The UL54 transcript was detected as early as 0.5 h, and peak expression was observed at 24 h. The UL54 gene was insensitive to the DNA polymerase inhibitor Ganciclovir (GCV) and the protein synthesis inhibitor Cycloheximide (CHX), both of which confirmed that UL54 was an immediate early gene.

**Conclusions:**

The DEV UL54 gene was expressed in a prokaryotic expression system and characterized for expression level, intracellular localization and gene kinetic class. We propose that these results will provide the foundation for further functional analyses of this gene.

## Background

Duck enteritis virus (DEV), a member of the alpha-herpes virus subfamily, induces an acute, hemorrhagic disease resulting in significant economic losses in waterfowl due to high mortality and low laying rates. As an alpha-herpes virus, DEV might share a similar genomic structure with Herpes simplex virus types 1 and 2 (HSV-1 and HSV-2), Pseudorabies virus (PRV), Varicella-zoster virus (VZV), Equine herpes virus types 1 and 4 (EHV-1 and EHV-4), and Bovine herpes virus type 1 (BHV-1). The genome is a linear double-stranded DNA molecule divided into a unique long region (UL) and a unique short region (US) flanked by an internal short repeat (IRS) and a short terminal repeat (TRS) [1]. During infection, the genes are expressed in a sequential cascade, termed immediate early (IE), early (E), and late (L) phases. The IE gene is immediately transcribed upon infection, without other proteins. The early gene is transcribed prior to viral DNA replication in an IE protein-dependent manner. Transcription of the late gene begins after the synthesis of DNA and viral protein is onset.

With the research of etiology, pathology, immunology, diagnostics, prevention and treatment, more information about DEV genes has been reported, except for UL54, which was predicted to encode a 51.75 kDa protein of 458 AA with 56 % homology to the corresponding HSV-1 protein ICP27. ICP27 is a conserved and multifunctional nuclear protein that translocates between the nucleus and the cytoplasm based on crucial nuclear localization (NLS) and nuclear export signal (NES) [2–8]. Furthermore, ICP27 has been implicated in viral replication, gene expression [9–16], apoptosis [17, 18] and host immunization reactions [19–22], all of which promote infection.

In the present study, UL54 was expressed as a tagged-protein with a molecular mass of apparent 66.0 kDa using an Escherichia coli expression system. Subsequently, we generated an UL54-specific antibody to analyze the expression level and intracellular localization of UL54 protein in DEV-infected cells. The transcript temporal class and susceptibility to CHX and GCV were characterized to demonstrate UL54 as an immediate early gene.

## Results and discussion

### The DEV UL54 protein was expressed in an E. coli expression system

**Fig. 1:**
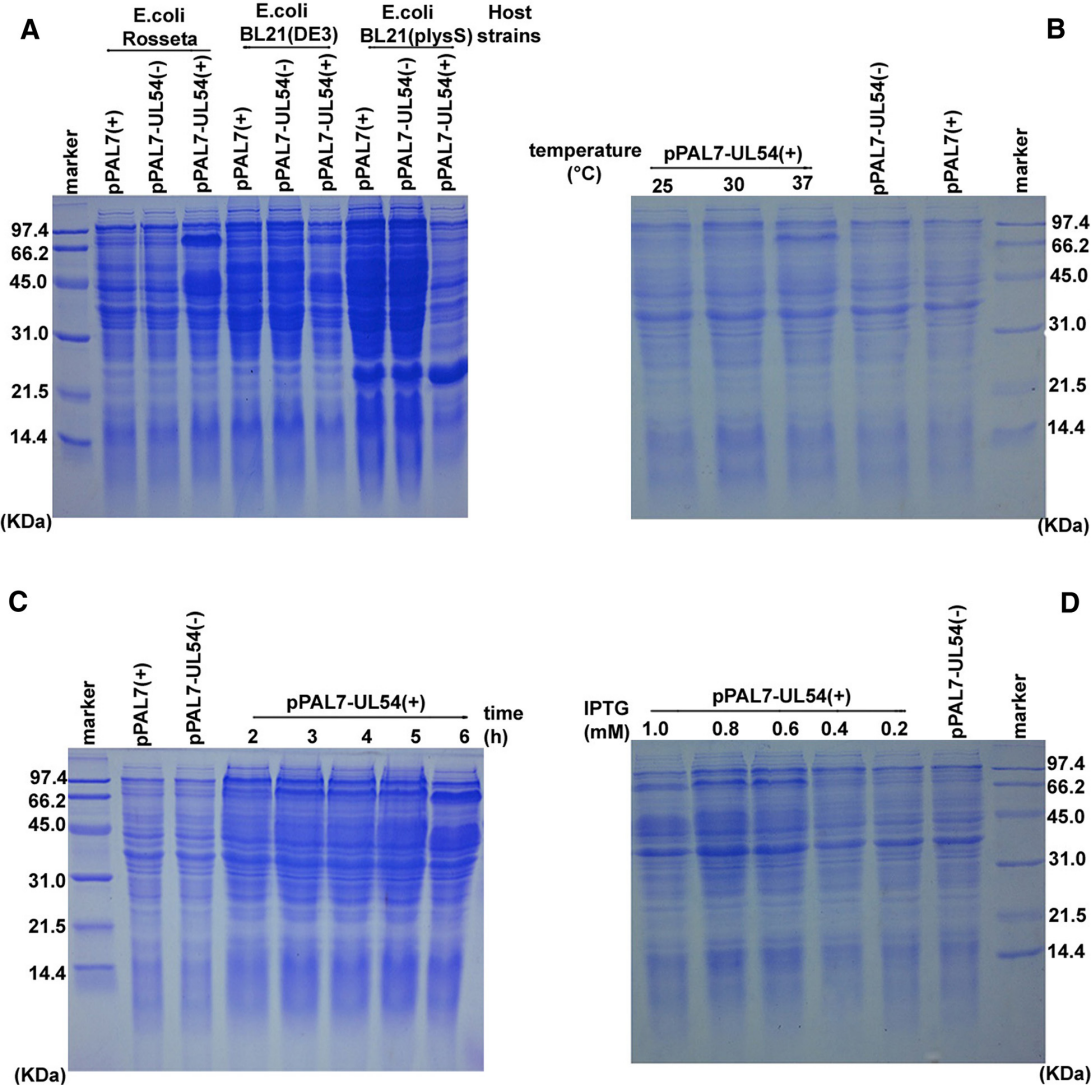
Analysis of UL54 protein expression. **a** The pPAL7 and pPAL7-UL54 were induced to express protein in E. coli Rosetta, BL21 (DE3), BL21 (pLysS). (−) and (+) represent incubation without and with IPTG, respectively. **b**, **c**, **d** UL54 protein was expressed at different temperatures (25, 30, and 37 °C), induction times (2, 3, 4, 5 and 6 h), and IPTG concentrations (0.2, 0.4, 0.6, 0.8, and l.0 mM)

The UL54 gene was cloned into vector pPAL7 and expressed under varying conditions, including different E. coli host cells, inducer concentrations, induction temperatures and induction durations (Fig. 1). The products were analyzed using SDS-PAGE, and there was no detectable UL54 gene expression in E. coli cells containing pPAL7 alone or non-induced pPAL7-UL54. However, a distinct band with a molecular mass of approximately 66.0 kDa (Profanity Exact-tag = 8.0 kDa) was visible when pPAL-UL54 expression was induced using IPTG in E. coli Rosetta at 37 °C. Furthermore, the expression of the UL54-Profinity Exact fusion protein was optimal when induced using 0.6 mM IPTG for 6 h.

**Fig. 2:**
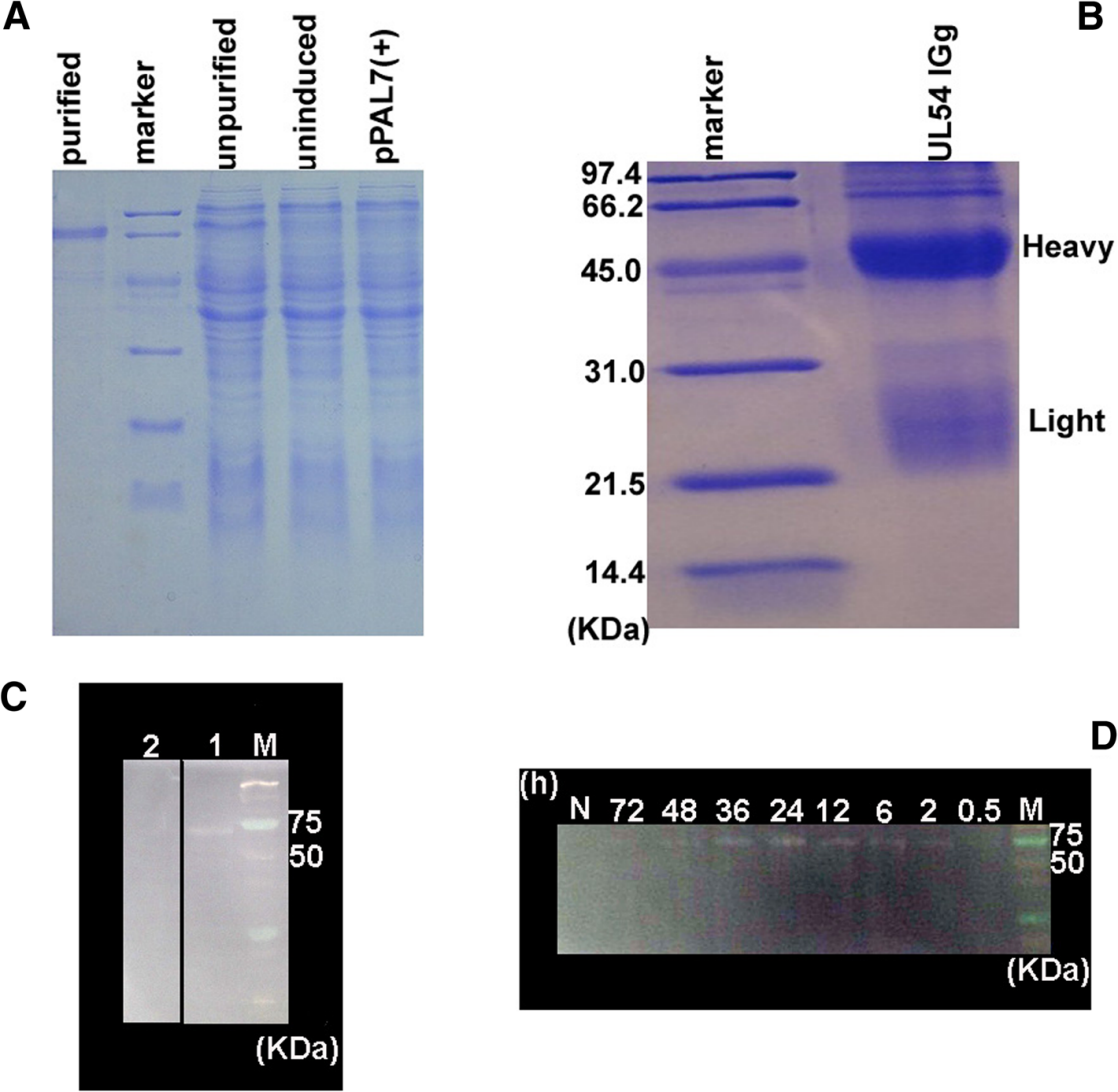
Preparation of anti-UL54 polyclonal antibody and UL54 expression analysis in DEV-infected cells. **a** UL54 protein was purified through gel and electric elution. **b** The anti-UL54 protein serum was cursorily purified using saturated ammonium sulfate. **c** Western blot analysis of the UL54 protein using rabbit anti-DEV-positive serum (lane 1) and rabbit negative control serum (lane 2). **d** Western blotting was used to analyze the lysates of DEV-infected DEFs harvested at different time points

Subsequently, the UL54 protein was expressed in E. coli Rosetta under optimized conditions and purified through gel and electric elution (Fig. 2a). The product was applied to generate the anti-UL54 polyclonal antibody (Fig. 2b), which was used for further studies. The protein was confirmed through Western blot analysis, and the results indicated that the rabbit anti-DEV antibody reacted with recombinant UL54 protein, revealing a specific band corresponding to a fusion protein of 66.0 kDa (Fig. 2c), consistent with the SDS-PAGE result showing a slightly higher than the predicted molecular mass of the UL54 protein. Notably, the amino acid composition might be responsible for the observed deviation.

### The DEV UL54 is an immediate early gene

Western blotting was used to detect DEV UL54 protein (Fig. 2d), which was initially detected at 2 h, and gradually increased expression was detected until 24 h, at which the highest expression level was observed.

**Fig. 3:**
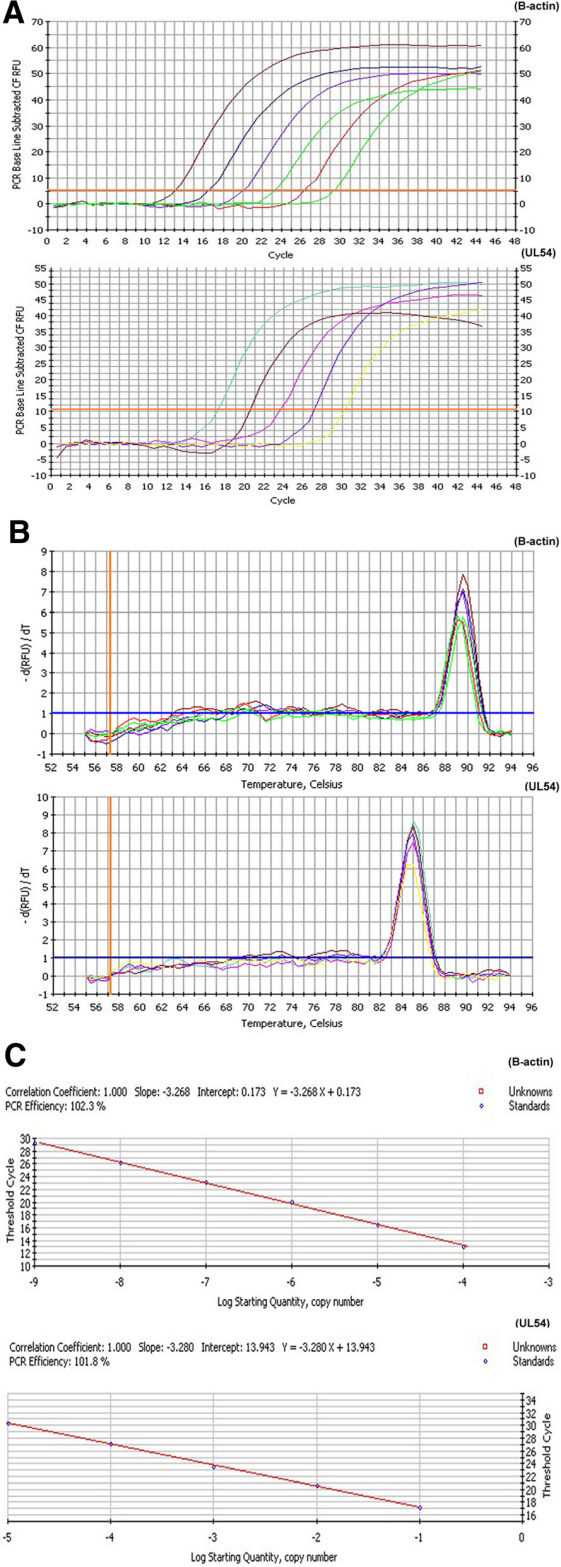
PCR amplification curves (**a**), melting curves (**b**) and standard curves (**c**) for B-actin and UL54

**Fig. 4:**
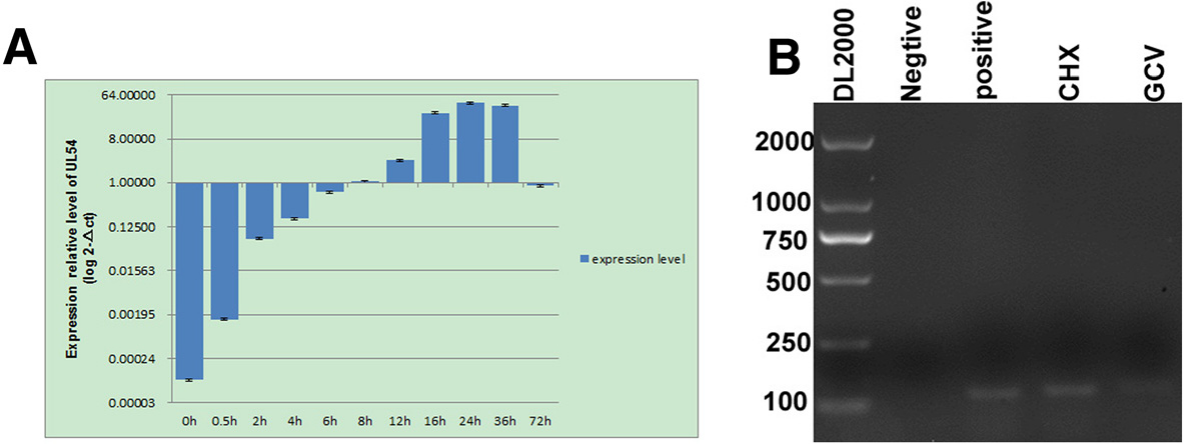
Transcription phase analysis of the UL54 gene. **a** Relative transcript levels of the DEV UL54 gene at different time points. **b** The pharmacological inhibition test showed that UL54 is an immediately early gene

Subsequently, we investigated the relative expression of the DEV UL54 gene in DEV-infected DEF cells at different time points using quantitative RT-PCR, the most sensitive technique currently used to analyze gene expression [23]. Standard curves, plotting plasmid copy number against the Ct values for the UL54 gene (Y = −3.280X + 13.943) and B-actin (Y = −3.268X + 0.173), were established to evaluate the efficiency of the assays (Fig. 3c), and the results were confirmed by the approximately identical amplification efficiency of the UL54 gene (101.8 %) and B-actin (102.3 %), with the correlation coefficients of 1.00 (Fig. 3a). Notably, the specificity of the primer sets was verified using a melting curve (Fig. 3b). Subsequently, total RNA was isolated, and after assessing the integrity, the RNA sample was reverse transcribed to cDNA, followed by RT-PCR and data processing to analyze the UL54 transcript levels. The results showed transcript expression as early as 0.5 h, and gradually increased, peaking at 24 h (Fig. 4a), confirming the results of the translation studies. Thus, these results suggested that the UL54 protein is an early gene product.

Moreover, we determined that UL54 is one of the several immediate-early genes based on the insensitivity of this gene to CHX and GCV (Fig. 4b). The positive and negative lanes represent uninfected and infected DEF cells without any treatment, respectively. Homologs of UL54 in other herpes viruses, such as PRV, BHV-1 and EHV-1, are synthesized with early kinetics [24–26].

The IE genes of DNA viruses encode regulatory proteins critical for viral infection. The five IE genes of HSV-1, namely ICP0, ICP4, ICP22, ICP27, and ICP47, have been identified, and among these [27, 28], ICP27 (UL54) is the most extensively studied for its potential regulation activity. However, an extreme degree of UL54 homology (29.5–43.1 %) had been observed in alpha-herpes virus counterparts, such as HSV-1, PRV, EHV-1, EHV-4, HSV-2, and VZV. High conservation and IE kinetics suggest that DEV UL54 might share a similar structure and regulatory function with HSV-1 ICP27, although this hypothesis requires additional studies.

### The DEV UL54 protein showed nuclear localization

**Fig. 5:**
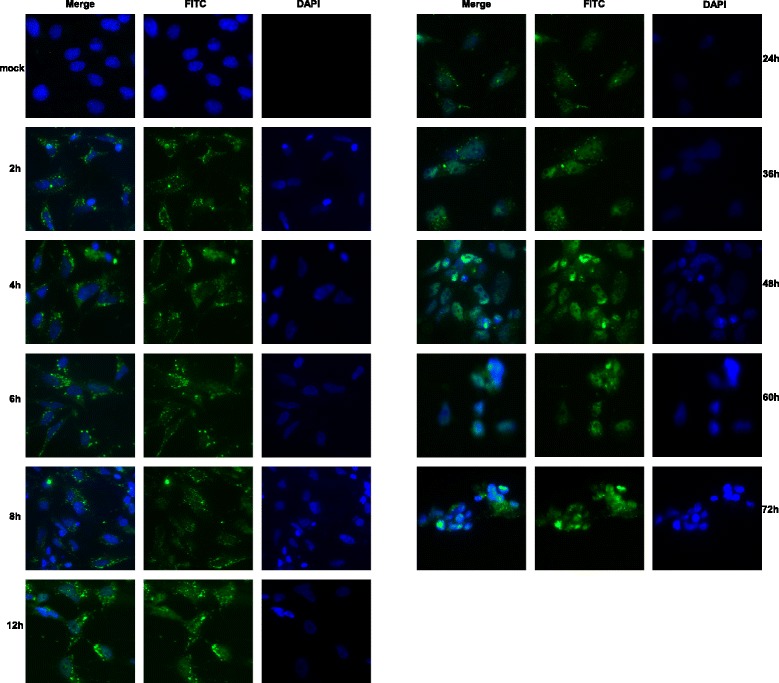
Dynamic intracellular localization of the UL54 protein during DEV infection

The intracellular distribution of DEV UL54 protein was confirmed through IFA using rabbit anti-UL54 serum. As shown in Fig. 5, UL54 protein-specific fluorescence was primarily located in the cytoplasmic region at 2, 4, and 6 h post-infection, and this fluorescence was gradually transferred to the nucleus. At 48 h post-infection, almost all UL54-specific fluorescence appeared in the nucleus, and subsequently, this signal became sparser and weaker following cytoplasmic disintegration. No fluorescence was observed in mock-infected cells. This result was consistent with the findings of previous bioinformatics analyses, showing that the UL54 protein is primarily localized to the nucleus [29]. The nuclear import of proteins is an essential step in regulating gene expression and the replication cycle, suggesting that DEV UL54 might have vital functions similar to HSV-1ICP27, which is primarily located in the nucleus and indispensable for viral infection [8].

## Conclusion

DEV UL54 was expressed as an approximately 66.0 kDa fusion protein in an E. coli expression system and subsequently identified as an immediate early gene that primarily localizes to the nucleus. More efforts are needed to elaborate the roles of UL54 in DEV infection.

## Methods

### Viruses and cells

DEV CHV strain was separated and preserved in the laboratory. Monolayer cultures of duck embryo fibroblast (DEF) cells were grown in Modified Eagle’s medium (MEM) supplemented with 10 % newborn calf serum (NBS) at 37 °C in a 5 % CO_2_ humidified incubator.

### Prokaryotic expression

The UL54 gene was amplified from the DEV genome and cloned into the pPAL7 vector, generating the recombinant plasmid pPAL-UL54, which was transformed into E. coli strains for subsequent protein expression [30, 31]. Subsequently, the colonies were picked, and expression was induced using isopropyl-B-thiogalactopyranoside (IPTG). The effects of different host cells (E. coli Rosetta, BL21 (DE3), and BL21 (pLysS)), inducer concentrations (0.2–1 mM), induction temperatures (25, 30, and 37 °C) and induction durations (2, 3, 4, 5, and 6 h) were examined to optimize the conditions for the highest level of UL54 protein expression. After IPTG induction cells were collected at different time point through cold centrifugation, and the products were determined through SDS-PAGE after disruption using cold sonication.

### Preparation of the polyclonal antibody

The UL54 protein was expressed and purified through gel and electric elusion, and used to generate a polyclonal antibody after purification through gel and electric elution. Approximately 0.5 mg of UL54 emulsified in complete Freund’s adjuvant and used to immunize eight rabbits through intradermal injections. Subsequent booster doses of 0.75 and 1.0 mg were prepared in incomplete Freund’s adjuvant, and the protein was administered after 2 and 3 weeks, respectively, using subcutaneous injections. Approximately 0.5 mg of UL54 protein was injected per rabbit. To collect the antibody, the rabbits were bled through an ear vein at 1 week after the last immunization. The antiserum was harvested, and a preliminary purification was conducted using saturated ammonium sulfate [32].

### Western blotting

The UL54 protein expression in E. coli Rosetta and DEF cells were detected using western blotting with rabbit anti-DEV and anti-DEV UL54 protein antibodies, respectively, as the primary antibodies [33]. A standard protocol was performed after the proteins were separated through electrophoresis on 12 % SDS-PAGE gels and transferred to polyvinylidene fluoride (PVDF) membranes using a semi-dry transfer cell apparatus. The membranes were blocked with PBS buffer containing 1 % BSA for 1 h and subsequently incubated with the diluted primary antibody for an additional 1 h. The membrane was washed for 30 min with 1× PBS containing 0.05 % Tween-20 and subsequently incubated with goat anti-rabbit HRP-labeled IgG secondary antibodies for 30 min. After washing, the membrane was developed using a DAB kit as previously described.

### Real Time PCR (RT-PCR)

Total RNA was isolated from DEV-infected DEF cells at different time points post-infection (0.5, 1, 2, 4, 6, 8, 12, 16, 24, 36, and 72 h) using Trizol, followed by DNase treatment during the RNA extraction. A sample of the total RNA was reverse-transcribed to cDNA using M-MLV (Moloney Murine Leukemia virus) reverse transcriptase after assessing the quality of the RNA using electrophoresis with 1 % agarose. Subsequently, real-time PCR was performed in a 20-μl-reaction volume containing 10 μL of SYBR Green Super Mix, 1 μLof each primer, 1 μL of cDNA, and 7 μL of ultrapure water. The thermal cycling procedure included initial denaturation for 1 min at 95 °C, followed by 45 cycles of denaturation at 95 °C for 5 s, annealing at 59 °C for 20 s and extension at 72 °C for 25 s. Triplicate experiments were performed to analyze gene expression of UL54 and B-actin, and the relative transcription level of the DEV UL54 gene was calculated using the 2^-ΔCt^ method simplified from the 2^-ΔΔCt^ method.

To evaluate the efficiency of each assay, standard curves were constructed, amplifying ten-fold serial dilutions of pPAL7-UL54 and pGM-T/B-actin [34, 35].

### Pharmacological inhibition reaction

Pharmacological inhibition was performed to confirm the DEV UL54 gene expression patterns. Total RNA was isolated from DEV-infected DEF cells incubated with GCV or CHX at 24 h post-infection and subsequently reverse transcribed into cDNA. The cDNA was used for subsequent PCR analysis, and the product was identified using a 1 % agarose gel.

### Indirect immune-fluorescence assay (IFA)

IFA was conducted using a standard procedure [36]. Briefly, DEV-infected DEF cells were plated onto coverslips and fixed with 4 % paraformaldehyde for 30 min at 0, 2, 4, 6, 8, 12, 24, 36, 48, 60 and 72 h post-infection. The fixed cells were permeabilized with 0.5 % Triton X-100 and incubated for 30 min in 5 % BSA at 37 °C. The anti-UL54 antibody and FITC-conjugated goat anti-rabbit IgG were used as primary and secondary antibodies, respectively, and the blots were sequentially incubated for 1 h. Subsequently, the cells were treated with 4’6-diamidino-2-phenylindole (DAPI) for 10 min to stain the nucleus. The images were captured using a fluorescence microscope after the coverslips were sealed with glycerin buffer onto glass slides.

## Abbreviations

BHV-1: Bovine herpes virus-1
CHX: Cycloheximide
DAPI: 4’6-diamidino-2-phenylindole
DEV: Duck enteritis virus
EHV: Equine herpes virus
GCV: Ganciclovir
HSV-1: Herpes simplex virus-1
ICP27: infectious cell protein 27
IE: immediate early
IFA: Indirect immunofluorescence
IPTG: isopropyl thiogalactoside
NES: nuclear export signal
NLS: nuclear localization signal
PRV: Pseudorabies virus
PVDF: polyvinylidene fluoride
RT-PCR: real-time quantitative reverse transcription PCR
SDS-PAGE: sodium dodecyl sulfate polyacrylamide gel electrophoresis
VZV: Varicella zoster virus
